# Novel Phlebovirus with Zoonotic Potential Isolated from Ticks, Australia

**DOI:** 10.3201/eid2006.140003

**Published:** 2014-06

**Authors:** Jianning Wang, Paul Selleck, Meng Yu, Wendy Ha, Chrissy Rootes, Rosemary Gales, Terry Wise, Sandra Crameri, Honglei Chen, Ivano Broz, Alex Hyatt, Rupert Woods, Brian Meehan, Sam McCullough, Lin-Fa Wang

**Affiliations:** Commonwealth Scientific and Industrial Research Organisation, Geelong, Victoria, Australia (J. Wang, P. Selleck, M. Yu, W. Ha, C. Rootes, T. Wise, S. Crameri, H. Chen, I. Broz, A. Hyatt, B. Meehan, S. McCullough, L.-F. Wang);; Department of Primary Industries, Parks, Water and Environment, Hobart, Tasmania, Australia (R. Gales);; Australian Wildlife Health Network, Mosman, New South Wales, Australia (R. Woods);; Duke–National University of Singapore Graduate Medical School, Singapore (L.-F. Wang)

**Keywords:** phlebovirus, bunyavirus, tick, zoonoses, Heartland virus, severe fever with thrombocytopenia syndrome virus, shy albatross, viruses, Australia

## Abstract

Recently discovered tick-borne phleboviruses have been associated with severe disease and death among persons in Asia and the United States. We report the discovery of a novel tick phlebovirus in Tasmania State, Australia, that is closely related to those zoonotic viruses found in Asia and North America.

Viruses in the family *Bunyaviridae* can infect animals and plants (*1*). The family is composed of 5 genera: *Orthobunyavirus*, *Phlebovirus*, *Nairovirus*, *Hantavirus*, and *Tospovirus* (*2*). The genus *Phlebovirus* contains known disease agents of animals, including humans, that can be carried by different vectors (e.g., phlebotomine sandflies, mosquitoes, and ticks) (*3*). In 2009, an outbreak of an acute febrile illness, known as severe fever with thrombocytopenia syndrome (SFTS), occurred in China. During an investigation of the outbreak, a previously unknown bunyavirus from the tick *Anaplasma phagocytophilum* was identified as the causative agent of SFTS (*4*). SFTS virus (SFTSV) has since been shown to be responsible for >150 human infections in 16 Chinese provinces and to have an associated death rate of ≈12% (*5*,*6*).

In June 2009, in northwestern Missouri, United States, 2 men from 2 geographically distant farms were hospitalized for fever, fatigue, diarrhea, thrombocytopenia, and leukopenia. Both men had been bitten by ticks 5–7 days before the onset of symptoms. A virus was isolated from the leukocytes of each patient and later identified as a novel phlebovirus by next-generation sequencing. The 2 viruses were highly related (98%, 95%, and 99% sequence identity for the small, medium, and large viral genome segments, respectively), indicating that the men were independently infected with the same virus. This new virus, named Heartland virus (HRTV), was most closely related to, but clearly distinct from, the SFTSV detected in China (*7*).

Subsequent to these disease events in China and the United States, fatal SFTSV infections were reported in humans in Japan (*8*) and Korea (*9*). Because similar viruses and human infections have been detected in 2 well-separated continents (i.e., Asia and North America), it is tempting to hypothesize that similar viruses may also exist in tick populations on other continents. We report the isolation and characterization of a phlebovirus from ticks in Australia that is similar to SFTSV and HRTV.

## The Study

In 2002, a disease outbreak occurred in a shy albatross (*Thalassarche cauta*) colony on Albatross Island, a small island in the Hunter Island Group in northwestern Tasmania, Australia. The disease in the albatrosses was characterized by weight loss and death. Serum samples and ticks (*Ixodes eudyptidis*) from healthy and affected birds were sent to the CSIRO (the Commonwealth Scientific and Industrial Research Organization) Australian Animal Health Laboratory in Geelong, Victoria, Australia, for diagnostic investigation. Several laboratory tests were conducted to detect potential viral pathogens involved in the disease event.

The ELISA or hemagglutination inhibition assay was used to test serum samples (N = 38) for antibodies against avian influenza virus, infectious bursal disease virus, fowlpox virus, and Newcastle disease virus. Neutralizing antibodies for Newcastle disease virus (titer of 64) were detected in 1 serum sample; otherwise, no antibodies against any of these suspected agents were detected.

Pooled tick homogenates (N = 5) were cultured on chicken embryos, chicken embryo fibroblast and skin cells, and Vero cells. No virus was detected in chicken embryos or in any of the chicken cell lines used. In contrast, cytopathic effect was observed on the third passage in Vero cell cultures inoculated with 2 tick pools (1 collected from diseased birds and the other from healthy birds).

**Figure 1:**
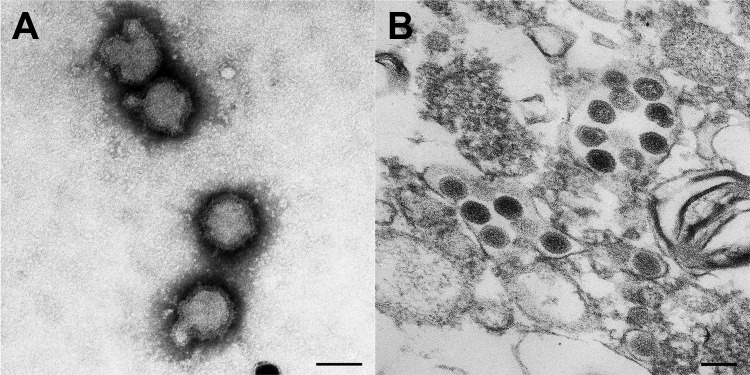
Electron microscopic examination results of a newly isolated virus, tentatively named Hunter Island Group virus, isolated from ticks collected from shy albatrosses, Tasmania, Australia. A) Negative-contrast staining of virions. B) Thin section of infected Vero cells showing the presence of viral particles. Original magnification ×100,000; scale bars represent 100 nm,

Electron microscopy examination revealed morphologically similar viral particles from both cultures; the morphologic features resembled those of bunyaviruses (Figure 1). However, testing with antibodies against a range of known bunyaviruses (orthobunyavirus, phlebovirus, and nairovirus) did not reveal any cross-reactivity. PCR analysis using bunyavirus-specific primers (*10*) also failed to detect any specific sequence from this newly isolated virus.

The investigation was put on hold until 2011 when the Australian Animal Health Laboratory established its next-generation sequencing capability. RNA extracted from partially purified virus grown in Vero cells was used for random PCR amplification. The resulting products were used for library preparation and subsequently sequenced by using the 454 GS FLX system (454 Life Sciences, Branford, CT, USA) according to standard protocols (*11*,*12*). In total, 6.9 Mb of sequence, consisting of 23,617 reads, was obtained and assembled into 722 contigs.

BLASTN (http://blast.ncbi.nlm.nih.gov/Blast.cgi) analysis at the nucleotide level showed no sequence identity to any of the known viruses in GenBank (data not shown). However, a protein-based BLASTX (http://blast.ncbi.nlm.nih.gov/Blast.cgi) search yielded 10 contigs showing protein sequence identity to SFTSV and some degree of sequence identity to several other members of the family *Bunyaviridae*. The virus was tentatively named Hunter Island Group virus (HIGV) after the location where the tick samples were obtained.

On the basis of the partial sequences derived from next-generation sequencing, primers were designed to obtain the rest of the genome sequences by using gap-filling PCR and 5′ and 3′ RACE methods previously developed by our group (*13*). The complete genome sequences of the large, medium, and small segments have been deposited in GenBank under accession numbers KF848980, KF848981, and KF848982, respectively.

**Figure 2:**
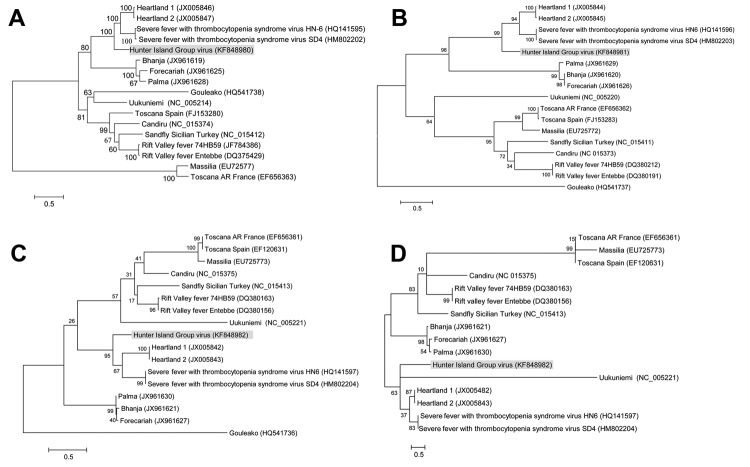
Phylogenetic trees of recently isolated bunyaviruses based on amino acid sequences of the polymerase protein (A) encoded by the large segment, the membrane glycoprotein polyprotein (B) encoded by the medium segment, and the nucleocapsid protein (C) and the nonstructural protein (D) encoded by the small segment of selected bunyaviruses. Maximum-likelihood trees were constructed by using MEGA5 (http://www.megasoftware.net/) with bootstrapping at 1,000 replicates. GenBank accession numbers are within parentheses next to the virus names. Scale bars indicate nucleotide substitutions per site.

The Table summarizes the genetic and sequence features of the genome segments and deduced proteins of HIGV and several selected phleboviruses. From the sequence comparison, it is evident that HIGV is a new member of the genus *Phlebovirus* and that it is most closely related to the newly discovered zoonotic members of the genus, SFTSV and HRTV. This finding was further confirmed by phylogenetic analysis based on nucleotide and protein sequences of all 3 genome segments (representative trees shown in Figure 2). Because HIGV was isolated from ticks collected from healthy and diseased birds we believed it was unlikely that the virus was the causative agent for the disease in the shy albatrosses. To further confirm this, we conducted ELISA and Western blot analysis using a recombinant HIGV nucleocapsid protein, which was expressed and purified from *Escherichia coli* by using previously described methods (*14*,*15*). Of the 38 serum samples tested, none produced positive readings in ELISA or Western blot. In addition, none were positive when tested by HIGV-specific quantitative PCR targeting the polymerase gene of the large segment.

**Table:** Summary of genomic and sequence features of Hunter Island Group virus and other selected phleboviruses*

Virus	Large segment			Medium segment			Small segment		
	Segment size, nt	Polymerase protein size, aa†		Segment size, nt	Glycoprotein size, aa†		Segment size, nt	Nucleocapsid protein size, aa†	Nonstructural protein size, aa†
HIGV	6,368	2,085		3,328	1,063		1,694	233	267
HRTV	6,368	2,084 (66)		3,427	1,076 (53)		1,772	245 (57)	299 (28)
SFTSV	6,368	2,084 (65)		3,378	1,073 (54)		1,744	245 (58)	293 (31)
BHAV	6,333	2,082 (35)		3,304	1,068 (26)		1,867	247 (39)	313 (17)
UUKV	6,423	2,103 (35)		3,229	1,008 (23)		1,720	254 (27)	273 (20)
RVFV	6,404	2,092 (34)		3,885	1,197 (21)		1,692	245 (35)	265 (18)

*HIGV, Hunter Island Group virus; HRTV, Heartland virus; SFTSV, severe fever with thrombocytopenia syndrome virus; BHAV, Bhanja virus; UUKV, Uukuniemi virus; RVFV, Rift Valley fever virus. †For proteins, the % amino acid sequence identity with cognate proteins of HIGV is shown in parentheses.

## Conclusions

We identified a novel tick-borne phlebovirus, HIGV, during the investigation of a disease outbreak among shy albatrosses in Tasmania. Genetic characterization showed that the virus is closely related to 2 newly discovered tick-borne zoonotic phleboviruses (SFTSV and HRTV) that were responsible for severe disease and death in humans in 4 separate countries in Asia and North America. However, with the current data alone, the particular disease event in the shy albatrosses could not be attributed to HIGV.

The findings from this study demonstrate the key role that a vigilant pathogen investigation has in any diagnostic assessment. The study findings also suggests that zoonotic phleboviruses genetically related to SFTSV, HRTV, and HIGV may be widely distributed in different parts of the world and that heightened international surveillance is needed to fully understand and appreciate the public health risk from these emerging viruses.
