# Correction: Vol. 20, No. 6

**DOI:** 10.3201/eid2008.C12008

**Published:** 2014-08

**Keywords:** errata, erratum, correction

In the first paragraph of the article Novel Phlebovirus with Zoonotic Potential Isolated from Ticks, Australia (J. Wang et al.), *Anaplasma phagocytophilum* should be replaced by *Haemaphysalis longicornis*. The article has been corrected online (http://wwwnc.cdc.gov/eid/article/20/6/14-0003_intro.htm).

